# Development and Psychometric Validation of the EDE-QS, a 12 Item Short Form of the Eating Disorder Examination Questionnaire (EDE-Q)

**DOI:** 10.1371/journal.pone.0152744

**Published:** 2016-05-03

**Authors:** Nicole Gideon, Nick Hawkes, Jonathan Mond, Rob Saunders, Kate Tchanturia, Lucy Serpell

**Affiliations:** 1 Research Department of Clinical, Educational & Health Psychology, University College London, London, United Kingdom; 2 Bedfordshire and Luton Eating Disorders Service, East London NHS Foundation Trust, Dunstable, United Kingdom; 3 Department of Psychology, Macquarie University, Sydney, Australia; 4 Centre for Health Research, Faculty of Medicine, Western Sydney University, Campbelltown, Australia; 5 Department of Psychological Medicine, Psychology and Neuroscience, Institute of Psychiatry, King’s College London, London, United Kingdom; 6 Eating Disorders Service, North East London NHS Foundation Trust, Essex, United Kingdom; United (Osaka U, Kanazawa U, Hamamatsu U Sch Med, Chiba U and Fukui U) Graduate School of Child Developmen, JAPAN

## Abstract

**Objective:**

The aim of this study was to develop and validate a short form of the Eating Disorder Examination Questionnaire (EDE-Q) for routine, including session by session, outcome assessment.

**Method:**

The current, 28-item version (6.0) of the EDE-Q was completed by 489 individuals aged 18–72 with various eating disorders recruited from three UK specialist eating disorder services. Rasch analysis was carried out on factors identified by means of principal component analysis, which in combination with expert ratings informed the development of an EDE-Q short form. The shortened questionnaire’s reliability, validity and sensitivity was assessed based on online data collected from students of a UK university and volunteers with a history of eating disorders recruited from a national eating disorders charity aged 18–74 (*N* = 559).

**Results:**

A 12-item short form, the Eating Disorder Examination Questionnaire Short (EDE-QS) was derived. The new measure showed high internal consistency (Cronbach’s α = .913) and temporal stability (ICC = .93; p < .001). It was highly correlated with the original EDE-Q (*r* = .91 for people without ED; *r* = .82 for people with ED) and other measures of eating disorder and comorbid psychopathology. It was sufficiently sensitive to distinguish between people with and without eating disorders.

**Discussion:**

The EDE-QS is a brief, reliable and valid measure of eating disorder symptom severity that performs similarly to the EDE-Q and that lends itself for the use of sessional outcome monitoring in treatment and research.

## Introduction

Eating disorders pose a serious challenge to mental health services due to their often chronic trajectory [1] and far-reaching psycho-social and medical consequences [2, 3]. It is therefore crucial to carry out appropriate psychological assessments and monitor progress throughout therapy so that care and treatment can be optimised. Evidence suggests that continuous collection and feedback of routine outcome measures leads to more positive treatment outcomes for patients [4–7]. Hence, public health authorities responsible for the delivery and regulation of mental health services increasingly demand the collection and reporting of patient outcome data [8].

The Eating Disorder Examination Questionnaire (EDE-Q) [9] is a 28-item, self-report measure derived from the Eating Disorder Examination (EDE) [10, 11], the latter being widely viewed as the “gold standard” in the assessment of eating disorder pathology [12]. The EDE-Q was developed to provide a self-report questionnaire that can approach the “gold standard” whilst being less onerous for patients [9]. It is widely used and is the only outcome tool for the assessment and monitoring of eating disorders recommended by the National Institute for Mental Health in England [13].

The EDE-Q’s psychometric properties have been extensively investigated in various study populations, including individuals with eating disorders receiving specialist treatment. The measure has been found to have strong psychometric properties in terms of internal consistency and test-retest reliability for both total scores and scores on subscales assessing four domains of eating disorder psychopathology (concerns about dietary restraint; concerns about eating; concerns about weight; concerns about shape) [14, 15, 16, 17]. Strong convergent validity between the EDE-Q and EDE has also been demonstrated in both clinical and general population samples [9, 12, 18].

There are, however, a number of problems with the EDE-Q. First, studies investigating the measure’s factor structure in various study populations have not supported the four-factor model entailed in the current subscales, while also failing to agree on an alternative factor structure [14, 19–21]. This raises the question of the appropriateness and utility of the existing subscales.

Second, despite general convergence in scores, people consistently score higher on the EDE-Q than on the EDE. This raises concerns about using these methods interchangeably [22]. Inconsistencies between the EDE and the EDE-Q have also been observed in the self-report assessment of certain eating disorder features, such as objective binge eating behaviours [9, 17, 18, 22–25], laxative use [9] and self-induced vomiting [26]. Discrepancies of this kind are typically taken to infer the superiority of interview assessment, although this is not necessarily the case [12, 25].

Third, although administration time for the EDE-Q is markedly reduced when compared with that for the EDE, the EDE-Q is still longer than ideal for use as a session-by-session outcome measure. Finally, the EDE-Q assesses the occurrence and frequency of symptoms over the past 28 days. This time frame makes the capture of identification of change from one week to the next problematic.

With these considerations in mind, the aim of the current study was to develop a short form of the EDE-Q, the EDE-QS, which can be used for sessional outcome measurement. We aimed for a measure with high reliability and construct validity, including strong positive correlations between the EDE-QS and the original EDE-Q, other measures of eating disorder pathology, and measures of comorbid psychopathology, and strong negative correlations with measures of quality of life. It was also hypothesised that the frequency of eating disorder behaviours would be comparable for the EDE-QS and the EDE-Q. A secondary aim of the study was to compare EDE-QS scores between people with and without a current eating disorder and thereby determine the measure’s sensitivity for differentiating between these subgroups.

### Study 1

The purpose of this study was to develop a short version of the EDE-Q, which could be used for sessional outcome monitoring.

## Methods

### Participants and procedures

This study obtained ethical approval from a National Health Service (NHS) ethics committee. EDE-Q data for 489 patients attending three UK Eating Disorders Services between April 2008 and January 2013 were included. Informed consent was not sought for this archival sample and patient data were anonymised and de-identified prior to analysis. One service had administered and collected EDE-Q version 6.0 (28 items, *N* = 297), whereas the other two services had used an older, 36 item version (*N* = 192). The main difference was the removal of frequency questions about subjective binge eating and diuretic misuse from version 6.0. Therefore, responses to the 36-item EDE-Q were mapped onto the latest version and the samples were combined.

The final sample included in- and outpatients. The majority was female (90.2%) and age ranged from 18 to 72 years (*M* = 31.5, *SD* = 11.5). The Global EDE-Q scale scores ranged from 1.4 to 6 (*M* = 4.2, *SD* = 1.2). Probable DSM-5 diagnoses [27] were derived from EDE-Q responses (see S1 Appendix for diagnostic methods employed). Sixteen percent of respondents were identified as probable anorexia nervosa (AN)—restrictive, 15% as probable AN–binge/purge subtype, 21% as probable bulimia nervosa (BN), 18% as probable binge eating disorder (BED) and 30% as probable other specified feeding and eating disorders (OSFED). Mean Body Mass Indices (BMI) were 14.23 (*SD* = 1.7) for AN–restrictive subtype, 14.79 (*SD* = 1.5) for AN–binge/purge subtype, 24.83 (*SD* = 7.8) for BN, 37.23 (*SD* = 13.8) for BED and 27.27 (*SD* = 13.6) for OSFED.

### Measures

#### EDE-Questionnaire

The current version of the EDE-Q (6.0) comprises 28 items. The 22 scaled items are categorised into four subscales: Restraint (5 items), Eating Concern (5 items), Shape Concern (8 items) and Weight Concern (5 items). Scores on each item range from “0” to “6”, with higher scores indicating higher symptom levels. As the subscales have varying numbers of items, subscale scores are calculated as average scores per item, also ranging from ‘‘0” to ‘‘6”. Based on Fairburn and Beglin’s (2008) instructions, the EDE-Q’s global score is commonly obtained by taking the mean of each of the subscales’ mean scores. The subscales vary in the number of items which they contain and therefore, some items are more heavily weighted than others. However, in view of concerns regarding the validity of the EDE-Q subscales [12, 14, 19], the global score for the current study was derived from the mean score of the 22 scaled questionnaire items, which also meant that all items received equal weighting. Items 13–18 of the EDE-Q elicit open responses to the frequency (number of times or days) of specific eating behaviours, such as objective binge eating (OBE), self-induced vomiting (SIV), laxative use (LAX) or excessive exercise (EX), over the last 28 days. These are not included in the subscale scores.

In this archival sample, Cronbach alpha for the global score was 0.90 and ranged from 0.70 for weight concern to 0.80 for shape concern.

### Statistical Analyses

The psychometric properties of the EDE-Q were explored using the Rasch model [28, 29]. Rasch analysis was chosen as it can be used to assess the appropriateness of a questionnaire’s rating scale, identify redundant or “misfitting” items for deletion and identify those items for retention that are sensitive to variance across the range of eating disorder severity. Due to the questionable validity of the EDE-Q’s subscales and inconsistent results with regards to number of factors and associated items in the literature (e.g., [19, 20]), an exploratory principal component analysis (PCA) was conducted first to derive the dimensions of the EDE-Q in our sample. Rasch analysis was carried out separately on each dimension to satisfy the model’s assumption of unidimensionality [30, 31]. A survey of mental health professionals obtained ratings on the importance of each EDE-Q item, i.e. how clinically meaningful scores or changes in score on each item are perceived to be. Information from the exploratory PCA, Rasch Modelling and expert survey was considered in conjunction to make decisions on the inclusion and exclusion of items.

#### Exploratory PCA

An exploratory PCA was carried out using SPSS 21, using oblimin rotation (oblique) to allow factors to correlate. Only the scaled EDE-Q items were included in the analyses. There was less than 5% of missing data for each scaled item, which were completely missing at random (Little’s MCAR test χ(741) = 754.79; p = .35). Imputations were made using the Expectation Maximisation method. Factors with eigenvalues above 1 were retained.

#### Rasch Analysis

The use of Rasch analysis was considered to be of particular importance as it is a good method for examining the appropriateness of a questionnaire’s rating scale and for identifying those items that are less useful as well as those that are more valuable for measuring a scale’s construct [32, 33].

Winsteps software was used (version Bond&FoxSteps [32]). The polytomous Rasch rating scale model was applied because the EDE-Q’s response scale is ordinal with seven response options.

1 Response Scale Properties

As a first step, the characteristics of the questionnaire’s rating scale were examined for each factor to assess whether their response categories were meaningful and informative. Rating scale criteria, as set out by Linacre [34], were examined:

- At least 10 responses should be present in each response category.
- There should be a regular distribution of responses across response categories.
- There should be a consistent increase of average measures with each category.
- Category thresholds should increase monotonically, ideally by at least 1.4 logits but no more than 5 logits [32]. This was also inspected visually by examining the probability curves for each factor. The individual curves should show distinct peaks for each category, indicating that each is the most probable response for some part of the eating disorder pathology [32].
- Category outfit mean square values should be less than 2.

Violation of these criteria prompted collapsing of categories. The rating scale diagnostics and probability curves of the collapsed models were then compared to the original to identify the optimal number of response categories [32]. Person and item separation indices were inspected to assess whether collapsing of response categories improved the reliability of persons and items. According to Bond and Fox indices should have values of at least 2 [32].

2 Model Fit

Items that have little predictive value and obtain unexpected ratings are said to misfit the model and introduce random variability into the data. Mean square infit and outfit values were used to assess this with acceptable values between 0.7 and 1.40 [32]. The item-measure correlation was also investigated. Values greater than 0.3 demonstrate that the item is sufficiently correlated to the overall concept or model [35]. Poorly fitting items were considered for deletion.

3 Redundant Items

Residual correlations between items within a scale were examined for local dependency, i.e. that responses to one item are dependent on or can be predicted by responses to another item, which implies item redundancy [33]. Residual item correlations that have values greater than 0.3 of the overall average of all correlations suggest local dependence [36]. Where this applied, deletion of one of the dependent items was considered.

4 Eating Disorder Severity

Each item’s difficulty estimate was calculated to select those items from each subscale that capture both mild and more severe eating disorder symptoms. Items that showed a strong overlap of difficulty (i.e., differences <0.20) were considered for deletion [37].

#### Expert Survey

A link to an online survey was emailed to mental health clinicians in the eating disorder field known to the authors. Experts were asked to categorise each EDE-Q item into “least important”, “very important—might be good to include” or “most important–needs to be included”. These categories were given values from 0–2 and summed up for each EDE-Q item. Their total scores were used to obtain an overall rating of importance for assessing clinical change. These could range from a minimum score of 0 to a maximum score of 20. Ten clinicians with at least six years expertise in working with people with eating disorders completed the survey. Professionals from different countries (including UK, USA, Canada and Australia) were invited to participate but unfortunately the country of residence was not recorded in the survey and details in this regard are therefore not available.

## Results

### Exploratory PCA

The exploratory PCA suggested a five-factor model with distinct and reliable factors (KMO = 0.874). This was further supported by the scree plot. Bartlett’s test of sphericity (5,289.84; *p <* .*001*) indicated data appropriate for PCA.

Factor I explained 33.01% of the total variance and Factor II added 13.04% of variance. Both consisted of six items. Factor III (4 items) explained 6.53% of additional variance. Factor IV added 5.34% of explained variance and consisted of two items only and Factor V (4 items) explained 4.98% of variance. None of the factors replicated the original EDE-Q’s subscales. Factor 2 and 3 resembled the original Shape Concern and Dietary Restrain subscale, respectively. However, they also contained items from other subscales. Factors 1, 4 and 5 differed substantially from the original subscales (see Table 1 for individual factor loadings). Factor 4, consisting of two items regarding secret and guilty eating, two criteria for a binge eating episode, resembled an indirect index of binge eating episodes.

**Table 1:** Summary of PCA, Rasch analysis, expert survey and diagnostic relevance.

	Item	Item content	PCA	Rasch analysis						Expert survey	Diagnostic relevance	Item selection	
			factor loading	item difficulty/severity	S.E.	infit MNSQ	outfit MNSQ	item total correl.	local item dependence *item (correl*.*)*	expert rating		included	rationale for inclusion/ exclusion & comments
**Factor I**													
	2	Long periods without eating	0.68	1	0.06	0.87	0.83	0.73		5	AN	✓	highest severity
	5	Empty stomach	0.7	0.13	0.05	0.89	0.79	0.71		5			not prioritised as medium severity
	6	Flat stomach	0.47	-0.6	0.06	1.2	1.06	0.6		5			similar severity as 7
	7	Preoccupation with food	0.55	-0.35	0.06	0.89	0.84	0.65		11		✓	highest expert rating & low severity
	21	Concerned to be seen eating	0.53	-0.14	0.05	1.03	1.11	0.6		5			similar severity as 7
	24	Upset to be weighed	0.52	-0.04	0.05	1.22	1.29	0.58		0			misfit; low expert rating
**Factor II**													
	11	Feeling of fatness	0.51	0.16	0.08	1.17	1.01	0.7	12 (0.36)	6			redundant; lower expert rating than 12
	12	Desire to lose weight	0.49	0.44	0.08	1.14	1.09	0.73	11 (0.36)	10		✓	highest expert rating & highest severity
	25	Dissatisfaction with weight	0.84	-0.11	0.09	0.83	0.81	0.72	26 (0.31)	5		✓	higher expert rating
	26	Dissatisfaction with shape	0.8	-0.33	0.09	0.82	0.85	0.68	25 (0.31)	4		(✓)	redundant; merged with 25
	27	Discomfort seeing body	0.8	-0.14	0.09	0.86	0.95	0.69	28 (0.33)	4			lower expert rating than 12 & 25
	28	Discomfort being seen	0.78	-0.02	0.08	1.08	1.16	0.67	27 (0.33)	1			redundant & low expert rating
**Factor III**													
	1	Limit amount of food	0.79	0.07	0.07	0.85	0.83	0.8		15	AN	✓	highest expert rating
	3	Exclude foods	0.81	0.37	0.07	0.81	0.78	0.83		10	AN		lower expert rating
	4	Dietary rules	0.75	0.31	0.07	1.05	1.02	0.79		10	AN		lower expert rating
	10	Fear of weight gain	0.5	-0.75	0.09	1.4	1.41	0.62		12	AN	✓	misfit but high expert rating & lower severity than 1
**Factor IV**													
	19	Eating in secret	0.74							5			no Rasch analysis possible; low expert rating
	20	Feeling guilty	0.51							5			no Rasch analysis possible; low expert rating
**Factor V**													
	8	Preoccupation with shape/weight	0.51	0.91	0.07	1.17	1.11	0.78		8			high expert rating, high severity
	9	Fear of losing control	0.63	0.26	0.07	1.32	1.27	0.71		9	BN		approached misfit
	22	Importance of weight	0.65	-0.59	0.09	0.78	0.77	0.71	23 (0.78)	9	AN/BN	✓	high expert rating, low severity
	23	Importance of shape	0.69	-0.58	0.09	0.69	0.65	0.72	22 (0.78)	7	AN/BN	(✓)	Redundant; merged with 22

S.E. = Standard Error

SIV = Self-induced vomiting

MNSQ = Mean square

LAX = Laxative use

correl. = correlation

EX = excessive exercise

AN = Anorexia nervosa

BN = Bulimia nervosa

BED = Binge eating disorder

incl. = include

### Rasch analysis

As the fourth factor comprised only two items, it was not included in a separate Rasch analysis [35, 38].

**Fig 1:**
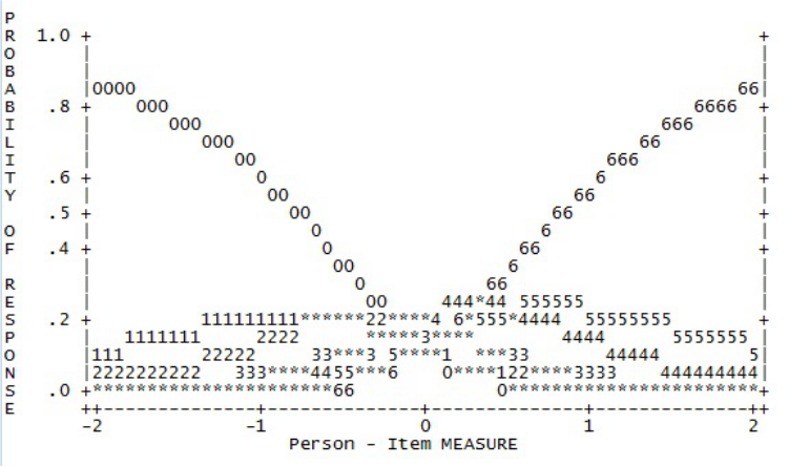
Response probability curve with original 7-point response options (Factor 5).

Rating scale diagnostics showed that responses across categories were not evenly distributed (e.g., rating scale category 2 held consistently less than 10% of responses) as respondents most commonly selected the most extreme response options (“no days” and “every day”). Further, all categories had disordered category thresholds, which was also clearly visible from the probability curves (see Fig 1 for an example). This implies that the given categories are not selected in a way consistent with the respondents’ severity of eating disorder. For example, more severely impaired persons may have selected response option three (13–15 days), whereas only mildly impaired people chose option four (16–22 days). The distinctions between the individual response categories might not be meaningful to participants and it suggests that there may be too many categories to choose from.

**Fig 2:**
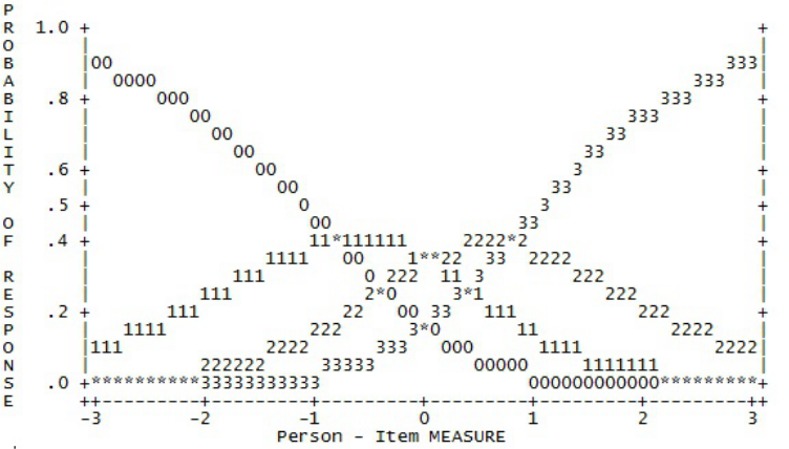
Response probability curve with collapsed 4-point response options (Factor 5).

To shorten the scale, response options 1 and 2, options 3 and 4, and options 5 and 6 respectively were combined. This produced a four-point response scale, which included values ranging from zero to three (0112233). This was applied across all factors as it was considered essential that all items of the short form use the same response scale. The revised four-point response scale demonstrated improved category thresholds, distribution of response frequencies and probability curves across all factors (see Table 2). All but one factor now showed ordered category thresholds and probability curves showed more distinct peaks (see Fig 2). Probability curves improved markedly but still showed respondents’ tendency to endorse the extreme points of the questionnaire, i.e. “no days” and “every day”.

**Table 2:** Rating scale diagnostics, reliability indices and visual inspection of probability curves for original and collapsed 4-point rating scale.

	Rating scale	Regular response frequency	Category thresholds	Outfit mean square	Person separation	Item separation	Probability curve
FACTOR 1	original	no	disordered	< 2.0	1.34	9.36	0 and 6 peak only
	4-point	improved	disordered	< 2.0	1.25	8.76	0, 1 and 3 peak
FACTOR 2	original	no	disordered	< 2.0	1.19	2.24	0, 4 and 6 peak
	4-point	improved	ordered	< 2.0	0.98	2.64	all peak
FACTOR 3	original	no	disordered	< 2.0	0.99	20.17	0 and 6 peak only
	4-point	improved	ordered	< 2.0	0.84	5.47	all peak
FACTOR 5	original	no	disordered	< 2.0	0.95	7.81	0, 4 and 6 peak
	4-point	improved	ordered	< 2.0	0.69	7.53	all peak

A high person separation index indicates that there is a good spread of responses, or in this case eating disorder pathology amongst the sample, which is likely to lead to consistent responding over time. Collapsing of response categories led to a slight reduction in the person separation indices for each factor. It was however decided to prioritise ordered thresholds over an already low person separation index. The item separation indices, which determine if items are responded to in the same way if given to a different sample, also reduced but remained above the recommended threshold of 2 [32].

### Expert survey

Ratings of items’ ability to indicate clinically significant change ranged from 0 to 15. Please refer to Table 1 for experts’ individual item ratings.

### Combination of methods

Table 1 summarises the results of the principal component analysis, Rasch model and expert survey for the scaled questionnaire items, alongside decisions and rationales for deleting individual items.

#### Frequency items

The frequency items were inspected in a similar fashion, investigating expert ratings and diagnostic relevance. Item 15 had high overlap in content with item 13 and 14. As the latter were rated higher by experts, item 15 was removed. Item 14 refers to a loss of control over eating. This has shown to be a better predictor of eating disorder pathologies than objective binge eating [39]. In order to have an independent item on perceived loss of control over eating [40] as well as a measure of objective binge eating, the order of items 13 and 14 was reversed. Respondents are therefore asked about perceived loss of control first, which is followed by a question on objective binge eating episodes. Items 16 and 17 refer to compensatory behaviours (i.e. taking laxatives and vomiting) and were combined into a single item.

Since the study aimed to develop a measure suitable for sessional outcome measurement, which is likely to be weekly, the response scale was recoded from a 28 day reference to seven days. To reduce missing responses and increase simplicity of coding of the frequency items, a Likert-scale response format was adopted. This resulted in the 12 item EDE-QS (see S2 Appendix), which, unlike the original EDE-Q, consists of a single scale.

## Discussion

The aim of study 1 was to develop a short form of the EDE-Q from questionnaire responses of people presenting to mental health services with a wide range of eating disorders. By combining statistical and expert based methods, we sought to develop a shortened version of the EDE-Q that is psychometrically and conceptually sound [41]. The exploratory PCA produced a five-factor model that did not replicate the original EDE-Q subscales, consistent with previous studies [14, 19, 20, 42].

Altering the response categories to a four-point rating scale, based on Rasch analysis, improved the functioning of the scale although a tendency of respondents to endorse the extreme points of the scale remained. Changing the reference time period of the scale to seven days was intended to improve accuracy of recall and permit evaluation of change over a shorter time frame. Combining statistical analyses and expert ratings resulted in the 12 item EDE-QS. The second study was conducted to evaluate the psychometric properties of the new measure, including its convergent and divergent validity, internal consistency, test-retest reliability and sensitivity in discriminating between people with and without eating disorders.

### Study 2

The aim of this study was to validate the EDE-QS, the newly developed short version of the EDE-Q.

## Methods

### Participants and procedures

This study obtained ethical approval from an NHS ethics committee. An email appeal providing a link to an online survey was sent out to all students of a London university. The same link was advertised on the website of Beat (Beating Eating Disorders), a charity supporting current and former sufferers of eating disorders. The link was further emailed to the charity’s email distribution list. All participants were given information about the scope and aims of the study, confidentiality and data protection. Their consent was assumed if they commenced the online study. The obtained data were anonymised and de-identified prior to analysis. The survey consisted of several online questionnaires as described below and was completed by 559 people. Of these, 54 (9.7%) self-reported that they currently suffered from an eating disorder. Twenty five of these were alerted to the study through the university email appeal and the rest through advertisement provided by the eating disorder charity. Probable eating disorder diagnoses in this group were assigned using DSM 5 criteria, based on their EDE-Q responses in combination with their BMI (see S1 Appendix for diagnostic criteria). Thirteen percent of respondents were identified as probable anorexia nervosa (AN)—restrictive, 4% as probable AN–binge/purge subtype, 15% as probable bulimia nervosa (BN), 19% as probable binge eating disorder (BED) and 50% as probable other specified feeding and eating disorders (OSFED). Of the 27 participants, who were given a probable diagnosis of OSFED, 24 (89%) had a global EDE-Q score of ≥ 2.3, the latter being a cut-point for ‘probable eating disorder case’ according to previous research in general population samples of young adult women [18].The majority of all respondents were aged between 18–34 years (*N* = 516; 92.5%) and female (*N* = 452; 80.9%). The majority (78%) were from White backgrounds. Most participants (88.9%) had completed higher education or basic university. Respondents were invited to provide their email address so that they could be recontacted a few days later to complete the EDE-QS only. Of 482 people who had left their email address and were contacted again, 335 (69.5%) completed the EDE-QS a second time.

### Measures

#### EDE-QS

The EDE-QS, as described in study 1, consists of 12 items, rated from zero to three. Cronbach’s alpha was 0.91.

#### EDE-Questionnaire (6.0)

As in study 1, the global EDE-Q score was derived from the mean score of the 22 scaled questionnaire items.Cronbach’s alpha for the EDE-Q’s global score was 0.96 in the current sample (study 2).

#### Clinical Impairment Assessment (CIA)

The CIA is a 16 item measure assessing impairment in psycho-social functioning secondary to eating-disorders. It enquires about personal, cognitive and social functioning on a four-point Likert scale. It has shown good psychometric properties and is useful in predicting eating disorder case status [2]. Cronbach’s alpha in the current study sample was 0.96.

#### Short Evaluation of Eating Disorders (SEED)

This is a brief eating disorder assessment instrument, consisting of 13 questions from which anorexia (ANTSI) and bulimia (BNTSI) severity indices can be derived. These range from a score of zero (“no symptoms”) to three (“extreme symptoms”). The SEED has been found to have acceptable construct and discriminative validity [43]. Cronbach’s alpha was 0.76 in the current study.

#### SCOFF Questionnaire

The SCOFF is a five item measure developed as a screening measure for use in primary care [44]. It consists of questions about key characteristics of anorexia and bulimia nervosa, which indicate self-induced vomiting, loss of control over eating, significant weight loss, perceived fatness and overvaluation of food. The name SCOFF is an acronym of words referring to these characteristics (i.e. **S**ick, **C**ontrol, **O**ne stone/month, **F**at and **F**ood). The SCOFF’s questions can be answered with a “yes” or “no” response. Two “yes” responses or more indicate that it is likely that the person is suffering from an eating disorder. The measure showed good validity in comparison to a clinical interview [45]. Cronbach’s alpha in the current study sample was 0.64.

#### Generalised Anxiety Disorder Questionnaire (GAD-7)

The GAD-7 is a brief seven item screening instrument for generalised anxiety disorder. Respondents rate each of seven anxiety symptoms on a four-point Likert scale over the past two weeks, ranging from “not at all” to “nearly every day”. The GAD-7 had good validity compared to independent mental health diagnoses, functional status measures, disability days and health care use [46] as well as good validity and reliability in the general population [47]. Internal consistency as measured by Cronbach’s alpha was 0.92 in the current study.

#### Patient Health Questionnaire (PHQ-9)

The PHQ-9 is a brief nine item questionnaire of depressive symptoms that is widely used as a standardised sessional measure for people receiving treatment in primary care. Responses are scored on a four-point, Likert-type scale, from “no days” to “every day”, indicating the frequency of depressive symptomatology during the past two weeks. The PHQ-9 has been found to have good psychometric properties [48]. Cronbach’s alpha in the current study sample was 0.91.

#### The World Health Organization Quality of Life (WHOQOL-BREF)

The WHOQOL-BREF is a brief, 26 item version of the original, 100-item, International Quality of Life Questionnaire (WHOQOL-100). The WHOQOL-BREF assesses individuals’ subjective evaluation of their quality of life in four domains, namely, physical health, psychological health, social relationships and environment. A higher score on this questionnaire indicates a better quality of life. It has been found to have good psychometric properties in a range of study populations and has been used in population-based studies of eating-disordered behaviour [49, 50]. Internal consistency as measured by Cronbach’s alpha was 0.77 for physical health, 0.73 for social relationships 0.78 for environment and 0.88 for psychological health.

### Statistical Analyses

The sample was divided into those who self-reported that they were currently suffering from an eating disorder and those who said they were not. This allowed the exploration of the EDE-QS’s performance in a general and clinical population. Chi square analyses were used to examine demographic differences, such as gender, age, ethnicity and level of education, between the two groups.

#### Reliability

Cronbach’s alpha coefficient was calculated to assess the homogeneity of the EDE-QS scale. An Intra Class Correlation coefficient (ICC) was computed between the overall EDE-QS at two administrations for temporal consistency, using a two way random model and type absolute agreement.

#### Validity

Spearman’s Rho co-efficient was used for correlational analyses between the EDE-QS and the other questionnaires to establish convergent validity as the data were non-normally distributed. The analyses were carried out separately for those respondents who reported a current eating disorder and those who did not. For comparative purposes, correlations between both, the EDE-QS and the EDE-Q, and other study variables were conducted for both “no ED” and “current ED” subgroups.

#### Frequency of Behaviours

The change in the EDE-QS response scale meant that it was not possible directly to compare frequencies for the behavioural EDE-Q items (e.g. binge eating) within a one-month period, so the kappa statistic was utilised to assess the chance-corrected level of agreement between the EDE-Q and the EDE-QS regarding the absence or presence of specific regular behaviours in one week, i.e. not at all vs at least once per week.

#### Sensitivity

The Mann Whitney-U test was applied to examine the difference between the EDE-QS scores of people with and without current eating disorders.

## Results

### Participants’ characteristics

Fewer men than women reported a current eating disorder (χ^2^ (1) = 11.45, p < .001). Participants with an eating disorder reported lower levels of education (χ^2^ (7) = 39.36, p < .001).

### Reliability

#### Internal consistency

Cronbach’s alpha coefficient showed that internal consistency for the EDE-QS was high (*α* = 0.913; *N* = 559). Item-total correlations ranged from 0.43 to 0.80 and item deletion would not have yielded a significant increase in reliability.

#### Test-retest reliability

Eating disorder symptom severity is not a stable trait and is expected to vary to an extent from time to time. However, a lot of change over a relatively short period was not anticipated either in the general population or in the clinical sample since they were not in treatment. Participants who completed the EDE-QS for the second time more than 14 days later were removed from the analyses, due to the greater possibility of a change in their eating disorder symptoms over time. The remaining sample (*N* = 328) returned the second EDE-QS on average 7.1 days later (range: 2–14 days). The ICC demonstrated a high and appropriate degree of temporal stability (*ICC* = 0.93; *p* < .001) (95% CI: 0.91, 0.94).

### Validity

#### Convergent validity

There was a significant and high correlation between the EDE-QS and the EDE-Q. The EDE-QS showed further significant and moderate to high positive associations with other measures of eating disorder pathology. Correlations with measures of general psychological functioning showed weaker significant correlations. There were negative correlations with the WHOQOL-BREF domains, other than the ‘environment’ domain for people with a current eating disorder (see Table 3). As is also apparent in Table 3, correlations between the EDE-QS and all other study measures were strikingly similar to those between the EDE-Q and these measures, and this was the case for both ‘no ED’ and ‘current ED’ subgroups.

**Table 3:** Convergent validity correlations for EDE-QS and EDE-Q.

	EDE-QS (no ED)	EDE-Q (no ED)	EDE-QS (current ED)	EDE-Q (current ED)
	*r*	*r*	*r*	*r*
*Convergent*				
**EDE-Q**	.91**		.82**	
**CIA**	.83**	.84**	.75**	.75**
**SCOFF**	.63**	.60**	.61**	.59**
**SEED ANTSI**	.64**	.65**	.46**	.39**
**SEED BNTSI**	.54**	.55**	.52**	.38**
**GAD-7**	.38**	.39**	.47**	.54**
**PHQ-9**	.50**	.50**	.61**	.6**
**WHOQOL-BREF**				
Physical health	-.34**	-.37**	-.34*	-.34*
Psychological	-.53**	-.56**	-.48**	-.58**
Social relationships	-.28**	-.28**	-.35**	-.37**
Environment	-.27**	-.26**	**-.11**	-.15

* p<0.05

** p<0.01

EDE-QS = Eating Disorder Questionnaire Short

ED = eating disorder

EDE-Q = Eating Disorder Questionnaire (6.0)

CIA = Clinical Impairment Assessment

SCOFF = acronym for Sick/Control/One stone in month/Fat/Food

SEED ANTSI = Short Evaluation of Eating Disorders Anorexia Nervosa Total Severity Index

SEED BNTSI = Short Evaluation of Eating Disorders Bulimia Nervosa Total Severity Index

GAD-7 = Generalised Anxiety Disorder Questionnaire

PHQ-9 = Patient Health Questionnaire

WHOQOL-BREF = The World Health Organization Quality of Life- Brief

### Frequency of Behaviours

The chance-corrected agreement between the EDE-QS and EDE-Q ratings of presence of at least one behaviour per week for people with self-reported eating disorders was excellent for days of binge eating (*kappa* = 0.7, *t* = 5.3, *p* < .001), laxative use or self-induced vomiting (*kappa* = 0.84, *t* = 6.24, *p* < .001), and excessive exercise (*kappa* = 0.89, *t* = 6.45, *p* < .001) [51].

### Sensitivity

People who reported a current eating disorder (*Mdn* = 17.5) scored higher on the EDE-QS than those who did not *(Mdn* = 5.0; *U* = 3209.5, *p* < .001).

## Discussion

Psychometric evaluation of the EDE-QS demonstrated excellent internal consistency and test-retest reliability. It showed very good convergent validity with the long version both, for people with and without an eating disorder.

The strong correlation with the CIA was to be as expected because the CIA measures psychosocial impairment secondary to eating disorder and, therefore, should be highly correlated with eating disorder symptom severity. The finding coincides with the developers’ validation study, which found a high correlation coefficient of .89 between the EDE-Q and the CIA [2], comparable to that obtained between the EDE-QS and the CIA and the EDE-Q and the CIA in people with and without an eating disorder in this study.

The EDE-QS showed a positive association with other measures of eating disorder pathology in both groups as hypothesised. The correlation between the EDE-QS and the SEED’s BN TSI and AN TSI was of medium strength. However, the SEED’s initial validation study [43] showed similarly low correlation (*r* = .32) with the Eating Disorder Inventory (EDI) [52], a well-established instrument for measuring symptoms of anorexia and bulimia nervosa. The relatively low correlation between the SEED and the EDE-QS and the EDE-Q also likely reflects the fact that both, EDE-QS and EDE-Q, are transdiagnostic measures, whereas the BNTSI and the ANTSI, like the EDI, relate specifically to bulimic and anorexic symptoms. Importantly, correlations between the EDE-QS and other measures of ED pathology were strikingly similar to those between the EDE-Q and each of these other measures and this was the case for both, ‘no ED’ and ‘current ED’ subgroups.

As would be expected, increased levels of problematic eating behaviours and attitudes were associated with increased feelings of anxiety, negative affect and higher levels of impairment in quality of life [53, 49].

The only exception was the domain of ‘environment’ in the quality of life measure as this did not show a negative association with the EDE-QS for people with eating disorders. It may be that environmental factors, such as financial resources and transport, may not necessarily be compromised with an increase in eating difficulties.

There was a high consistency between the EDE-Q and the EDE-QS with regards to reporting behaviours typical of eating difficulties, i.e. binge eating, self-induced vomiting, laxative use and excessive exercise. As would be expected, the EDE-QS differentiated between people who reported suffering from an eating disorder and those who do not.

Demographic information showed that men were underrepresented in the group with eating disorders. This is consistent with findings from previous research [54–56]. People without eating disorders in this study were also more likely to have obtained higher levels of formal education. However, this was to be expected as the majority of people without eating disorders were recruited from a university population.

## General Discussion

This study used a multi-method approach to develop and validate a short form of the EDE-Q, the 12 item EDE-QS. To this end, two studies were conducted. In the first study a combination of PCA, Rasch modelling and expert ratings, was applied to identify the most relevant items for inclusion in a shortened version. Rasch analysis was also used to improve the existing rating scale categories and make them more suitable for a weekly outcome measure. The second study examined the new measure’s psychometric properties.

Rating scale analysis identified problems with the original EDE-Q’s response scale. It highlighted that respondents did not make full use of the scale and failed to differentiate meaningfully between the intermediate response categories. This may have arisen due to differences and difficulties in calculating the exact number of days within one month on which specified experiences or behaviours occurred. Differences between EDE-Q and EDE ratings have been thought to be due to problems in retrieval strategies [18]. During the EDE interview memory prompts are given by using a calendar, which may enhance recall [57]. It is unclear which retrieval strategies are applied during the self-report questionnaire; it is however likely that there is a high cognitive demand which may impact on accuracy. The EDE-QS has a four-point rating scale referring to the past week only, which is likely to reduce cognitive demand. Referring to the past week also helps to obtain more accurate diagnostic criteria, i.e. symptoms being present at least once per week [27]. By providing scaled response options instead of inviting handwritten responses for the frequency items, it is also more likely that missing or unreadable data can be minimised.

Given the aforementioned problems with the original EDE-Q’s response categories, it may be useful to revise them and to consider a reduction in the number of response options. This is likely to improve the accuracy of people’s responses and to make it more user-friendly.Overall, the EDE-QS correlated as expected with other measures for people with and without eating disorders. High correlations between the short-form and the original EDE-Q suggest that the most relevant and informative items have been retained in the shortened version. Aardoom et al. [42] established that the EDE-Q’s global score is a valid indicator for a person’s level of eating disorder severity. It is therefore likely, that the EDE-QS total score is similarly sensitive, however this needs to be further investigated.

### Clinical implications

Due to its brevity and revised response categories, the EDE-QS lends itself to being used as a weekly measure permitting ongoing progress monitoring, which has been shown to improve patients’ outcomes [4] and is increasingly implemented in mental health settings. Based on Fairburn, Cooper, Shafran and Wilson’s transdiagnostic protocol for the treatment of eating disorders [57], positive outcomes are more likely if change in eating behaviours and symptoms occurs within the first six weeks of starting therapy and should therefore be a focus of attention.

Data quality is a challenge for services. A shorter questionnaire reduces the burden on patients and staff, whilst session-by-session monitoring permits use of the last measure as the ‘end’ measure, both of which could improve completion rates. Continuous outcome monitoring referring to the past week can therefore provide valuable information to the clinician. Change or indeed the absence of change in symptoms may provide useful material for therapeutic discussions and could help the clinician shape the intervention, which researchers view as the key element for the observed benefits of progress monitoring [5, 58].

A progress monitoring instrument like the EDE-QS could also be utilised in research studies to identify moments of change, including sudden or early changes, in eating attitudes and behaviours and in time series experimental designs. A shorter tool, if it is seen as more proportionate, is also advantageous for assessing eating disorder symptom severity in clinical settings where eating disorder may be a feature but not the primary focus, such as personality disorder or bariatric services.

Further research, however, will be needed to establish the EDE-QS’s sensitivity to change over time in different clinical settings and in different subgroups of patients. Other brief questionnaires have been developed to assess eating disorders, such as the SEED, SCOFF, Eating Disturbance Scale (EDS-5 [59]) or the Eating Disorder Examination–Screen (EDE-S [60]). However, these measures are either screening instruments and do not cover the clinical construct of eating disorder pathology or focus specifically on anorexia and bulimia nervosa, which make their transdiagnostic use as sessional outcome measures problematic. The EDE-QS was designed specifically for continuous outcome monitoring. It would be of interest to know whether the new measure might also be useful as a screening measure, namely, in identifying probable cases of eating disorders in general population samples and/or primary care. However, validation of its use for this purpose would require data from both the short form and from a diagnostic measure such as the EDE interview [9, 18].

### Limitations of study and future research

Several limitations of the current study should be noted. First, data bearing on participants’ ethnicity was not available, hence it is unclear whether and how the psychometric properties of the new measure might differ for ethnic subgroups. Further, this study was conducted within an adult population and the questionnaire’s suitability in adolescents remains yet to be investigated.

The sample size of consulted experts in this study was small, and a convenience sampling method was used, which may have limited generalisability. According to Okoli and Pawlowski [61], opinions gathered by ten people are sufficient to obtain general agreement. However, as the expert opinion influenced item selection, a larger sample may have been desirable.

For the psychometric analysis, the sample was divided into people who self-reported an eating disorder and those who did not, and probable eating disorder diagnoses were derived from their EDE-Q scores. The use of diagnostic assessments or interviews for this purpose would have been preferable. In addition, the number of people who identified themselves as having an eating disorder was relatively small. It would therefore be desirable to continue the EDE-QS’s psychometric evaluation using a larger eating disorder sample and investigate the measure’s performance in different diagnostic groups.

Future studies should also assess the EDE-QS’s ability to detect change over the course of treatment. It would be desirable to establish clinically significant change indices or cut-off points for the EDE-QS to differentiate between non-clinical and clinical impairment in eating disorders. This could guide clinicians with regard to treatment planning and prioritising.

Strengths of this study included its empirical derivation from the EDE-Q and indirectly the EDE, grounding it in the extensive clinical and research bases of these measures. It also used a large and diverse archival sample with regards to eating disorders (with the exclusion of avoidant\restrictive food intake disorders). In addition, the current study used robust methods by combining Rasch analyses with factor analytic techniques and expert views.

In sum, the EDE-QS appears to be a promising short-form of the EDE-Q suitable for use in sessional outcome and monitoring assessment of individuals with eating disorders receiving specialist treatment. Further research will be needed to continue its psychometric evaluation in larger samples and diagnostically different groups.

## Supporting Information

S1 Appendix Criteria to arrive at probable DSM-5 diagnosis. (DOCX)

S2 Appendix Eating Disorder Examination Questionnaire—Short (EDE-QS). (DOCX)

S1 Raw Data Study (XLSX)

S2 Raw Data Study (XLS)
