# Correction: Development and Psychometric Validation of the EDE-QS, a 12 Item Short Form of the Eating Disorder Examination Questionnaire (EDE-Q)

**DOI:** 10.1371/journal.pone.0207256

**Published:** 2018-11-05

**Authors:** Nicole Gideon, Nick Hawkes, Jonathan Mond, Rob Saunders, Kate Tchanturia, Lucy Serpell

## Notice of republication

An incorrect version of S2 Raw Data Study was published in error. This article was republished on October 24, 2018 to correct for this error. Please download this article again to view the correct version.
