# Supplementary material for: Development and Psychometric Validation of the EDE-QS, a 12 Item Short Form of the Eating Disorder Examination Questionnaire (EDE-Q)
Source: PLoS One. 2016 May 3;11(5):e0152744. doi: 10.1371/journal.pone.0152744 (PMC4854480; doi:10.1371/journal.pone.0152744)
Supplement: S1 Appendix — (DOCX) [file pone.0152744.s001.docx]

| **Diagnosis** | **BMI** | **Compensatory behaviour** | **Frequency over past 28 days** |
| --- | --- | --- | --- |
| Anorexia Nervosa- restrictive | < 17.5 | No regular binge eating and purging episodes | Less than once/week |
| Anorexia Nervosa – binge/purge subtype | < 17.5 | Binge eating OR purging episodes | At least once/week |
| Bulimia Nervosa | >18.5 | Binge eating AND purging episodes | At least once/week |
| Binge Eating Disorder | >18.5 | Binge eating episodes | At least once/week |
| Other Specified Feeding or Eating Disorder (OSFED) | Remaining cases | | |
