# Supplementary material for: Development and Psychometric Validation of the EDE-QS, a 12 Item Short Form of the Eating Disorder Examination Questionnaire (EDE-Q)
Source: PLoS One. 2016 May 3;11(5):e0152744. doi: 10.1371/journal.pone.0152744 (PMC4854480; doi:10.1371/journal.pone.0152744)
Supplement: S2 Appendix — (DOCX) [file pone.0152744.s002.docx]

**EATING DISORDER EXAMINATION QUESTIONNAIRE -**

**SHORT (EDE-QS)**

Name: ____________________ Date: ___________________ Weight: _______ Height: ________

**ON HOW MANY OF 0 1-2 3-5 6-7**

**THE PAST 7 DAYS…. days days days days**

1. Have you been deliberately trying to limit the

amount of food you eat to influence your weight or 0 1 2 3

shape (whether or not you have succeeded)?

1. Have you gone for long periods of time

(e.g., 8 or more waking hours) without eating anything 0 1 2 3

at all in order to influence your weight or shape?

1. Has thinking about food, eating or calories

made it very difficult to concentrate on things you 0 1 2 3

are interested in (such as working, following

a conversation or reading)?

1. Has thinking about your weight or shape made

it very difficult to concentrate on things you are 0 1 2 3

interested in (such as working, following a

conversation or reading)?

1. Have you had a definite fear that you might 0 1 2 3

gain weight?

1. Have you had a strong desire to lose weight? 0 1 2 3
2. Have you tried to control your weight or shape

by making yourself sick (vomit) or taking laxatives? 0 1 2 3

1. Have you exercised in a driven or compulsive

way as a means of controlling your weight, shape 0 1 2 3

or body fat, or to burn off calories?

1. Have you had a sense of having lost control 0 1 2 3

over your eating (at the time that you were eating)?

1. On how many of these days *( i.e. days on which*

*you had a sense of having lost control over your* 0 1 2 3

*eating*) did you eat what other people would

regard as an unusually large amount of food in one go?

**OVER THE PAST 7 DAYS … Not at all Slightly Moderately Markedly**

1. Has your weight or shape influenced how you 0 1 2 3

think about (judge) yourself as a person?

1. How dissatisfied have you been with your weight 0 1 2 3

or shape?

**Derived from the EDE-Q, © Fairburn and Beglin, 2008**
